# Ambiol Prevents Changes in the Functional Characteristics of Mitochondria Under Hypoxia

**DOI:** 10.3390/ijms27083589

**Published:** 2026-04-17

**Authors:** Irina V. Zhigacheva, Natalya I. Krikunova, Elena M. Mil, Ludmila I. Matienko, Marina A. Yakovleva, Alexander N. Goloshchapov

**Affiliations:** Emanuel Institute of Biochemical Physics, Russian Academy of Sciences, St. Kosygin, 4, Moscow 119334, Russia; kric@mail.ru (N.I.K.); elenamil2004@mail.ru (E.M.M.); matienko@sky.chph.ras.ru (L.I.M.); lina.invers@gmail.com (M.A.Y.); golan@sky.chph.ras.ru (A.N.G.)

**Keywords:** ROS, mitochondria, LPO, fatty acids, antioxidants, benzimidazole, Ambiol

## Abstract

Oxidative stress occurs when there is an excess of reactive oxygen species (ROS) in the cell, primarily produced by mitochondria. Excess ROS trigger membrane lipid peroxidation (LPO), cause mitochondrial swelling, and release proapoptotic proteins into the cytoplasm, which can lead to apoptosis. It is assumed that antioxidants that reduce excessive ROS formation by mitochondria can increase the body’s resistance to stress factors. We investigated the effects of hypoxia and the antioxidant Ambiol (2-methyl-4-dimethylaminomethylbenzimidazole-5-ol dihydrochloride) on the functional characteristics of mitochondria, which were assessed by measuring lipid peroxidation intensity using spectrofluorimetry, mitochondrial membranes fatty acid composition using chromatography, mitochondrial morphology using atomic force microscopy, and respiration rate using polarography. Injecting mice with Ambiol at a dose of 10^−6^ mol/kg for 5 days prevented the stress-induced activation of lipid peroxidation, a decrease in the unsaturation index of C_18_ and C_20_ fatty acids in mitochondrial membranes, and swelling of these organelles. The drug also increased the efficiency of oxidative phosphorylation during the oxidation of NAD-dependent substrates. Furthermore, Ambiol increased the lifespan of mice by 3.0–4.0 times under various types of hypoxia. Ambiol’s ability to maintain initial (control) levels of C_18_ and C_20_ unsaturated fatty acids appears to protect against stress-induced mitochondrial dysfunction.

## 1. Introduction

The search for new protective drugs capable of maintaining the homeostasis of physiological systems of living organisms under stressful conditions is a critical task in biomedicine. In particular, under oxidative stress, bioantioxidants are promising candidates for the role of such protective agents [1,2,3]. Long-term or intense exposure to stress factors leads to the accumulation of reactive oxygen species (ROS) inside cells, originating from mitochondria. ROS can interact with polyunsaturated fatty acids, integral components of mitochondrial membrane lipids, initiating lipid peroxidation (LPO) [4,5,6]. Furthermore, ROS induce oxidation of the SH group of Cys-56 (Cysteine-56) in the ATP/ADP antiporter, causing the opening of permeability transition pore (PTP) [7,8]. This leads to a disruption of the osmotic balance between the mitochondrial matrix and the intermembrane space, leading to mitochondrial swelling, cytochrome C release, and, potentially, the induction of apoptosis [9,10]. It can be assumed that antioxidants, by reducing the generation of ROS by mitochondria, should exhibit protective properties against oxidative stress.

Some benzothimidazole derivatives are known to possess antioxidant and antiradical properties [11,12,13,14]. These derivatives and imidazole derivatives are structural components of many natural compounds and modern medications [15].

**Figure 1:**
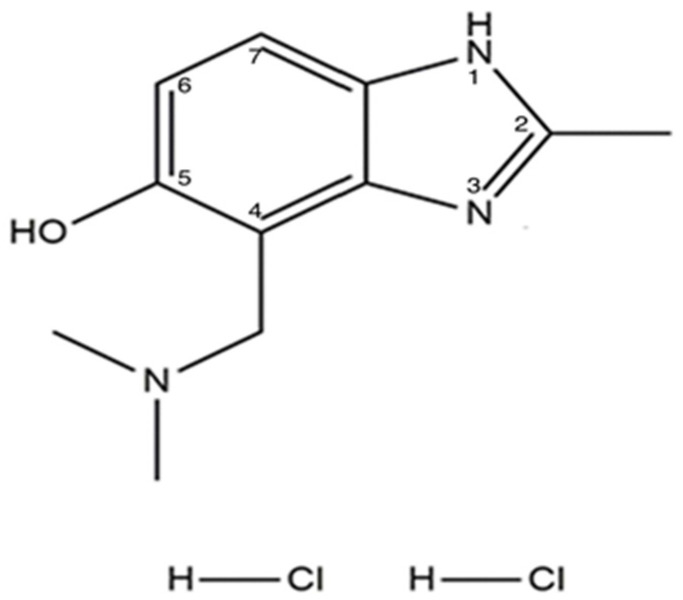
Ambiol.

The plant growth regulator Ambiol, (2-methyl-4-dimethylaminomethylbenzimidazole-5-ol-dihydrochloride), is a benzimidazole derivative (Figure 1):

Ambiol (AMB) has been shown to improve the plant’s tolerance to adverse environmental conditions. It induces a non-specific defense mechanism in agricultural plants against fungal and bacterial pathogens and decreases the radionuclide concentration in agricultural products [16]. Treatment of plants with Ambiol accelerates growth and increases their productivity, which may be associated with increased levels of cytokinins and auxins, as well as increased photophosphorylation and photosynthesis [17].

Ambiol also has application in pharmacology, increasing the effectiveness of antitumor chemotherapy and reducing its toxicity [18]. The use of Ambiol in chronic heart failure at a dose of 17 mg/kg leads to increased myocardial contractility [19]. It is assumed that Ambiol enhances plant resistance to stress factors through its influence on mitochondrial functional characteristics [20], and may exhibit similar adaptogenic properties in animals. Indeed, prolonged exposure to stressors leads to excessive generation of reactive oxygen species (ROS) by mitochondria, disrupting cell metabolism. We assumed that antioxidants, such as Ambiol, could reduce the generation of ROS by mitochondria and thereby reduce LPO, increasing the body’s resistance to stressors. Considering the crucial role of mitochondria in the body’s stress response [21], we aimed to investigate the effect of this drug on the functional state of mouse liver mitochondria under stress conditions, particularly hypoxic conditions. We assumed that this drug could be used to prevent ischemia-induced mitochondrial dysfunction [22].

## 2. Results

### 2.1. Inhibition of Lipid Peroxidation by Ambiol—Study on “Aging” Mitochondria

In all the experiments disclosed below, AMB was dissolved in double-distilled water. The biological activity of antioxidants varies depending on their dose [23]. Therefore, it was necessary to determine whether Ambiol exhibits antioxidant properties and at what concentrations they are manifested. To solve this problem, we used a model of mitochondrial “aging”. (Mitochondria, containing 2–3 milligrams of protein, were placed in a 0.5 mL medium, which contained 65 mM KCl, 10 mM HEPES, and 1 mM KH_2_PO_4_, pH 7.4. The mitochondria were incubated for 20 to 25 min at room temperature.) Lipids were extracted from the mitochondria containing 3–5 mg of protein using a 2:1 (*v*/*v*) mixture of chloroform and methanol. The ratio of mitochondria (mg) to chloroform-methanol mixture (*v*) was 1:10.

**Figure 2:**
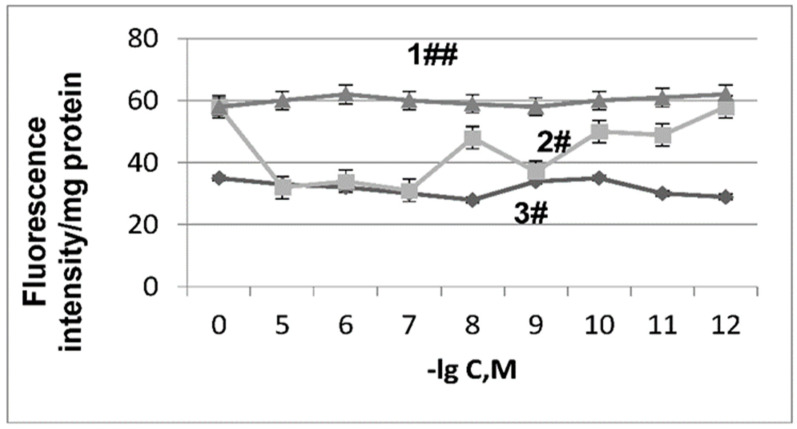
The effect of “aging” and Ambiol on the fluorescence intensity of LPO products (Schiff bases). The X-axis corresponds to (−lg) concentrations of Ambiol (AMB), the Y-axis to fluorescence intensity of LPO products in arbitrary units/mg of protein. Legend: 1—“aging” of mitochondria (without additives of Ambiol); 2—“aging” of mitochondria + AMB; 3—control. Fluorescence was measured in 10 mm quartz cuvettes. The excitation wavelength was 360 nm, and the emission wavelength was 410–560 nm. The symbol # denotes the difference between two curves (2 and 3), and the symbol ## denotes the difference between curve 1 and curves 2 and 3. Designations # refer to *p* < 0.05 and ## refer to *p* < 0.01.

The “aging” of mitochondria was accompanied by the activation of lipid peroxidation, the intensity of which was recorded by the fluorescence of Schiff bases, which are formed between the amino groups of proteins and aldehydes (4-hydroxy-2, 3-nonenal or malondialdehyde). The fluorescence of these end products of lipid peroxidation (Schiff bases) in mitochondrial membranes of “aging” mouse liver mitochondria increased by 1.7 times (Figure 2, curve 1).

The addition of Ambiol into the mitochondrial incubation medium caused a dose-dependent reduction in fluorescence levels. The most effective concentrations, which reduced the fluorescence intensity of lipid peroxidation products in the mitochondrial membranes to control levels (Figure 2, curve 3) were 10^−5^, 10^−7^, and 10^−9^ M.

A decrease in LPO intensity after the introduction of Ambiol into the mitochondrial incubation medium suggests that the drug has anti-stress properties. These properties were investigated in experiments using an acute hypobaric hypoxia model (AHH). This model was chosen because acute hypobaric hypoxia causes mitochondrial dysfunction, leading to an increase in reactive oxygen species (ROS) levels and to the activation of lipid peroxidation in cells and tissues [24].

### 2.2. Activation of Lipid Peroxidation in the Membranes of Mouse Liver Mitochondria Under Conditions of Acute Hypobaric Hypoxia (AHH)

Acute hypobaric hypoxia was modeled in mice using a glass pressure chamber at a low pressure of 230.40 mm Hg, corresponding to an altitude of 9000 m above sea level. Mice were exposed to this altitude for 5 min. Before the stress exposure, experimental mice received intraperitoneal injections of Ambiol at a dose of 10^−6^ mol/kg for 5 days, while control mice received saline solution according to the same schedule and volume.

**Figure 3:**
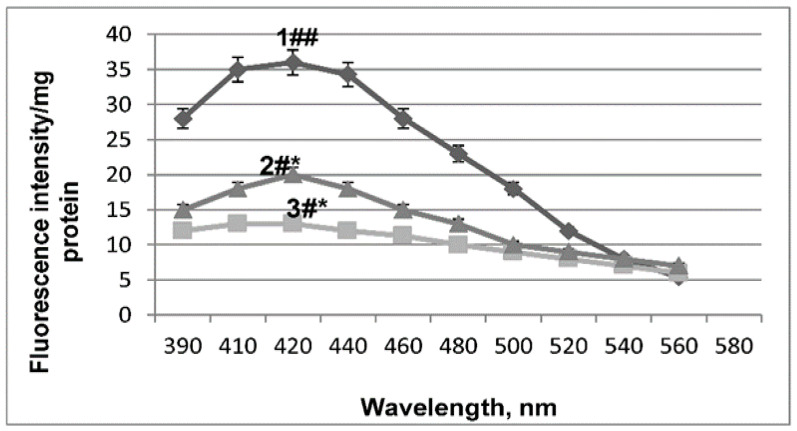
Changes in fluorescence spectra of mitochondrial lipid peroxidation products (1 mg of protein) in mouse liver membranes under AHH and Ambiol treatment. 1—AHH; 2—AHH + 5-day injections of 10^−6^ mol/kg Ambiol to mice; 3—control. Fluorescence spectra were measured as shown in the legend to Figure 2. Designations * and # refer to *p* < 0.05 and ## refer to *p* < 0.01.

Acute hypobaric hypoxia (AHH) caused LPO activation, which was accompanied by a 2.8-fold increase in the fluorescence intensity of lipid peroxidation products (Figure 3, curve 1). These findings are consistent with previous studies by M. Marzorati [24].

Five-day injection of Ambiol to mice prevented the activation of lipid peroxidation (Figure 3, curve 2); the fluorescence intensity of lipid peroxidation products was practically at the control level (Figure 3, curve 3).

### 2.3. Study of the Mouse Liver Mitochondria Volume Using Atomic Force Microscopy

**Figure 4:**
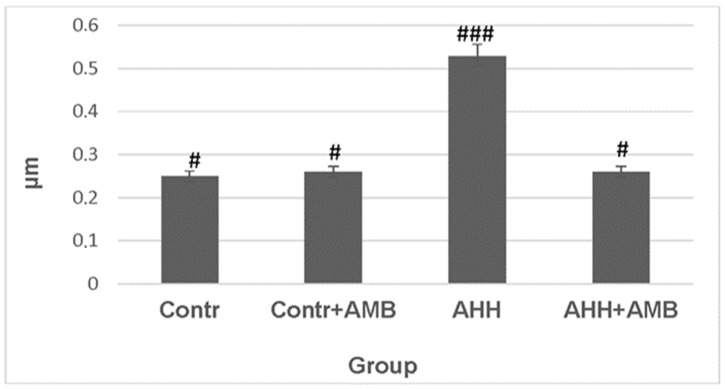
The values of average mouse liver mitochondrial volume (using atomic force microscopy) under acute hypobaric hypoxia and with Ambiol injections. Designations ### means that the probability of difference between column 3 and columns 1,2,4 is *p* < 0.001, and # the probability of difference between columns 1,2,4 is 0. Designations # refer to *p* < 0.05 and ### refer to *p* < 0.001.

The increased intensity of lipid peroxidation in mitochondrial membranes under acute hypobaric hypoxia conditions caused swelling of these organelles. The SOLVER P47 SMENA device operating at a frequency of 150 kHz in semi-contact mode was used for the study of mitochondrial volume. The analysis was performed using an NSG11 cantilever with a radius of curvature of 10 nm. Some geometric parameters of the mitochondrial image were determined using Image Analysis software Number 3.4x. The cross-section of the images was taken at a height of 30 nm. AFM images of liver mitochondria in mice subjected to acute hypobaric hypoxia showed a volume of 0.534 ± 0.003 μm^3^ (Figure 4, Table 1). The average mitochondrial volume in the control group of animals was 0.243 ± 0.003 μm^3^.

Ambiol injected into the control group of animals did not result in any changes in mitochondrial volume. However, Ambiol effectively protected mitochondria from volume changes (swelling) under acute hypobaric hypoxia conditions. Moreover, mitochondrial size was comparable to that of the control group of mice, further supporting the hypothesis that the drug has anti-stress properties.

### 2.4. Fatty Acid Composition of Liver Mitochondrial Membranes Under Conditions of Acute Hypobaric Hypoxia

The activation of lipid peroxidation probably should have affected the fatty acid (FA) composition of the total mitochondrial lipid fractions. When studying the effect of lipid peroxidation on the fatty acid composition of mouse mitochondrial membranes, one animal from each group was sacrificed simultaneously at the same time (10 a.m.). Studies were conducted on 10 mice from each group. The fatty acid composition of mouse liver mitochondrial membranes was studied by gas chromatography and chromatographic mass spectrometry. Fatty acid methyl esters (FAMEs) were obtained by acid methanolysis of mitochondrial membrane lipids [25,26]. They were extracted using hexane, and the resulting solutions were analyzed.

**Figure 5:**
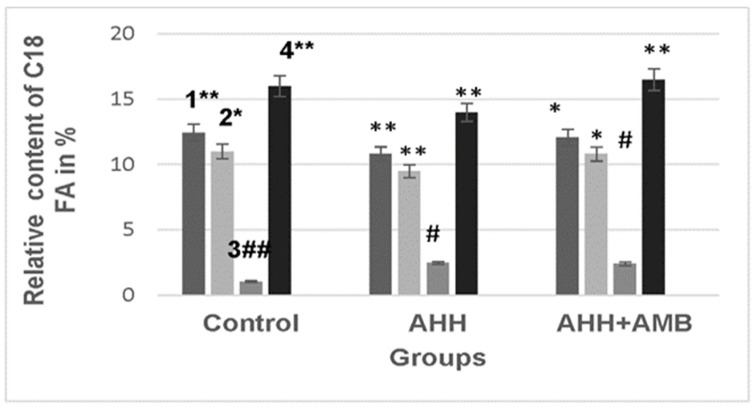
The relative content of C18 fatty acids in mouse liver mitochondrial membranes under acute hypobaric hypoxia, with and without Ambiol injection. 1—18:2ω6; 2—18:1ω9;. 3—18:1ω7; 4—18:0. Designations * and # refer to *p* < 0.05, while ** and ## pertain to *p* < 0.01.

Exposure to acute hypobaric hypoxia led to changes in the fatty acid composition of mitochondrial membranes. In particular, changes were observed in the levels of C_18_ fatty acids, with a decrease in the level of 18:2ω6 by 13% (Figure 5, column 1) and a decrease in 18:1ω9 by 14% (Figure 5, column 2). At the same time, there was a decrease of 9% in the unsaturation index of C_18_ and a reduction of 5% in their unsaturation coefficients.

**Figure 6:**
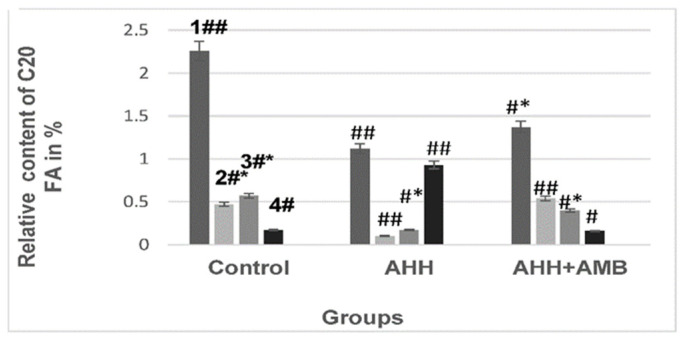
The relative content of C_20_ fatty acids in mouse liver mitochondrial membranes during acute hypobaric hypoxia, with and without Ambiol injection. 1—20:3ω3; 2—20:2ω6; 3—20:1ω9; 4—20:0. Designations * and # refer to *p* < 0.05, while ## pertain to *p* < 0.01.

The C_20_ fatty acids content has been modified. The content of 20:4ω6 in the mitochondrial membranes under AHH remained almost unchanged; however, the pool of 20:3ω3 decreased by 50.5% (Figure 6, column 1), 20:2ω6-by 80% (Figure 6, column 2), and 20:1ω9-by 70% (Figure 6, column 3).

Concurrently, there was a 19% decrease in the C_20_ fatty acid unsaturation index and a 30% decrease in the unsaturation coefficient. Ambiol injections to mice prevented changes in the fatty acid composition of liver mitochondrial membranes. Note that although the 20:3ω3 content of mitochondrial membranes increased, it was still 39% lower than the control level. The fact that Ambiol prevented changes under stress suggests its potential anti-stress effects.

### 2.5. Oxidation Rates of NAD-Dependent Substrates by Liver Mitochondria Under Hypoxic Conditions and When Treated with Ambiol

Changes in the mitochondrial membrane fatty acid profile affect the activity of mitochondrial respiratory chain enzymes, which is assessed by the rate of electron transport in the mitochondrial respiratory chain during oxidation of NAD-dependent substrates. The rate of respiration of mouse liver mitochondria was measured polarographically using a LP-7 polarograph (Czech) with a Clark electrode. AHH causes a decrease in maximum oxidation rates of NAD-dependent substrates (Table 2), consistent with data from Prikhodko V.A [27].

The incubation mixture consisted of: 0.25 M sucrose, 10 mM Tris-HCl (pH 7.5), 2 mM KH_2_PO_4_, 5 mM MgSO_4_, and 10 mM KCl. It was further supplemented with: 200 μM ADP, 10^−6^ M FCCP (carbonylcyanide-*p*-trifluoromethoxyphenylhydrazone), 4 mM glutamate, and 1 mM malate. The legend: AHH—acute hypobaric hypoxia; V_2_—substrate oxidation rate; V_3_—substrate oxidation rate in the presence of ADP; V_4_—the rate of oxidation at rest (substrate oxidation rate upon exhaustion of ADP).

There was a decrease in the maximum oxidation rate of NAD-dependent substrates by almost 30%, which was accompanied by a 1.6-fold decrease in respiratory control rate (RCR). Injection of 10^−6^ mol/kg Ambiol to mice for five days prevented changes in the bioenergetic characteristics of mitochondria. The oxidation rate of NAD-dependent substrates in the presence of FSSP or ADP remained unchanged compared to the control values. Notably, the RCR remained stable compared with the RCR observed in the group of animals exposed to AHH. The drug’s ability to restore mitochondrial bioenergetic functions under AHH conditions likely stems from its protective effect on the physicochemical properties of mitochondrial membranes. The presence of AMB in the incubation medium of intact mouse liver mitochondria resulted in elevated rates of oxidation for NAD-dependent substrates. The observed increase in oxidation depends on the AMB concentration in the mitochondrial incubation medium (Table 3).

The drug stimulated the oxidation of NAD-dependent substances by liver mitochondria, achieving a 1.2 to 1.5-fold increase in the presence of the uncoupler FCCP (carbonylcyanide-*p*-trifluoromethoxy-phenylhydrazone). Additionally, it stimulated an increase in the rate of oxidation of these substrates by a 1.3 to 1.6-fold in the presence of ADP, with optimal concentrations of 10^−5^ and 10^−6^ M maximizing the increase in oxidation rates and oxidative phosphorylation efficiency.

Thus, the anti-stress properties of the drug are apparently related to its ability to influence mitochondrial function. A five-day administration of AMB at a dose of 10^−6^ mol/kg increases lifespan by 3.0–4.0 times under various types of hypoxia. The drug also increased dynamic performance: the duration of swimming with a load in mice increases more than twofold (Table S1, Supplementary Materials).

## 3. Discussion

Based on the data obtained, it can be concluded that the anti-stress activity of AMB is due to its ability to prevent the activation of lipid peroxidation. Under stress conditions, including various types of hypoxia, the amount of ROS in the cell increases, and mitochondria play a key role in this process [22]. Reactive oxygen species serve as signaling molecules, helping the body adapt to unfavorable conditions. However, if they are formed in excess, they can cause damage to cellular components. Excessive ROS formation leads to lipid peroxidation, especially of phospholipids, and the oxidative modification of proteins and nucleic acids. Unsaturated fatty acids, especially those with a chain length of 18, 20, and 22 carbon atoms, undergo oxidative modification in mitochondrial membranes [28]. It has been shown that such modification of the lipid composition of the membrane fraction of these organelles leads to the disruption of lipid-protein interactions and, as a consequence, to mitochondrial dysfunction. Firstly, these changes also lead to a significant decrease in mitochondrial function, namely, a 25% decrease in the activity of complex I of the respiratory chain [28,29,30,31]. Indeed, under conditions of acute hypobaric hypoxia, we observed a 30% decrease in the maximum oxidation rates of NAD-dependent substrates. This probably led to a subsequent disruption of complex IV of the respiratory chain, which is observed along with mitochondrial swelling and the partial release of cytochrome C into the cytosol [32]. Antioxidants, by preventing the excessive formation of reactive oxygen species by mitochondria, support the energy function of these organelles, increasing the body’s resistance to stressors [33]. Thus, antioxidants are broad-spectrum adaptogens [33,34,35,36]. It should be noted that some benzimidazole derivatives exhibit antioxidant activity [11,12,13,37], which likely prevents the development of oxidative stress. Indeed, we demonstrated that Ambiol prevented the activation of lipid peroxidation, i.e., inhibited the development of oxidative stress. Due to these properties, AMB has found pharmacological applications: it exhibits a hepatoprotective effect and is also used in the treatment of chronic heart failure [19]. It should be noted that the drug exhibits its protective properties in the same concentration range as the mitochondria-targeted antioxidants MitoQ and SkQ [38]. The advantage of antioxidants targeting mitochondria is that they accumulate in the mitochondrial matrix due to the presence of the membrane potential, i.e., they accumulate and reduce the generation of ROS at the sites of their formation [39]. A disadvantage is their instability and low bioavailability. Damaged mitochondria exhibit a reduced ability to absorb mitochondria-targeted antioxidants. It is noteworthy that the intracellular activity of mitochondrial ROS differs from that of cytosolic ROS, as they perform specific intracellular functions that can be disrupted by the careless use of mitochondria-targeted antioxidants [40]. Unlike these drugs, Ambiol is a stable and water-soluble compound, which is an undoubted advantage.

Our findings on the effect of Ambiol on the bioenergetic characteristics of mitochondria, when added to the incubation medium, could indicate a direct effect of the drug on mitochondrial respiratory chain enzymes. However, we suggest that these effects are likely due to the drug’s influence on the physicochemical properties of mitochondrial membranes.

It is also worth noting that Ambiol is a plant growth regulator [16], increasing plant resistance to abiotic stress not only by reducing mitochondrial ROS generation, but also by activating antioxidant enzymes such as guaiacol peroxidase, ascorbate peroxidase, and increasing the total ascorbic acid concentration [41].

At the same time, we have found that this drug increased the resistance of animals to hypoxia. By preventing lipid peroxidation, Ambiol protected unsaturated C_18_ fatty acids, notably linoleic acid (a crucial component of cardiolipin) [39], from oxidative damage. Likely, this protection maintained the optimal performance of mitochondrial respiratory chain supercomplexes [42] and preserved energy metabolism, thereby enhancing the body’s resilience to stressors. It is possible that Ambiol, as in plants and animals, is involved in increasing the activity of antioxidant enzymes and the expression of antioxidant genes. Therefore, further research into the mechanisms of Ambiol’s protective action is planned.

## 4. Materials and Methods

### 4.1. Materials

Ambiol was first synthesized in the 1990s at the Emanuel Institute of Biochemical Physics of the Russian Academy of Sciences and has found wide application in crop production as a plant growth regulator. The trademark for Ambiol is registered under certificate number 258534. For the experiment, Ambiol was synthesized by staff of the Institute in January 2024.

The experiment used reagents of the following companies:

Methanol, chloroform (Merck, Darmstadt, Germany), sucrose, Tris, (Sigma, St. Louis, MO, USA), BSA (free from fatty acids) (Sigma, St. Louis, MO, USA), ADP, FCCP mM glutamate, malate (Sigma, St. Louis, MO, USA), HEPES (MP Biomedicals, Eschwege, Germany), potassium carbonate, (Merck, Darmstadt, Germany), hexane (Panreac, Barcelona, Spain), acetyl chloride (Acros Organics, Geel, Belgium).

### 4.2. Fluorescence Measurements

A fluorescence method was used to determine the intensity of lipid peroxidation (LPO) [43]. This method quantifies the fluorescence emitted (at an excitation wavelength of 360 nm) by Schiff bases formed during the reaction of protein amino groups with aldehydes, in particular malondialdehyde and 4-hydroxy-2,3-nonenal, which are the end products of LPO. Lipids were isolated from mitochondria containing 3–5 mg of protein using a chloroform-methanol mixture in a 2:1 (*v*/*v*) ratio. Fluorescence measurements were performed using a FluoroMax-Horiba Yvon GmbH spectrofluorimeter (Horiba Scientific, Longjumeau, France).

### 4.3. Experiments with Animals and Biomaterials

For the experiments, Balb/c male mice were selected. These animals were 3 months old and weighed 25–30 g each, and the groups consisted of 15 individuals. AMB was administered intraperitoneally at a concentration of 10^−6^ mol/kg over five days. The preparation involved dissolving AMB in double-distilled water and each dose given to the mice was 0.2 mL. The last drug administration of the drug took place 45 min before the stress exposure. All mice received a standard diet.

A control group of mice was given double-distilled water in amounts identical to those of AMB solutions in order to determine the effects of the solvent. It was confirmed that double-distilled water did not affect mitochondrial function itself, indicating that any observed effects were solely due to the AMB solution. The study was conducted in accordance with the principles of biomedical ethics outlined in the 1964 Helsinki Declaration and its subsequent amendments (European Convention for the Protection of Vertebrate Animals Used for Experimental and Other Scientific Purposes (ETS No. 123, Strasbourg, 1986) and approved by the Ethics Committee of N.M Emanuel Institute of Biophysical Chemistry of the Russian Academy of Sciences (Moscow, Protocol No. 2 dated 8 February 2024).

Differential centrifugation, as described in [44], was used to isolate mitochondria from mouse liver. This involved an initial 10 min centrifugation at 600× *g*, followed by a second 10 min centrifugation at 9000× *g*. The resulting mitochondrial pellet was then re-suspended in a solution containing 0.25 M sucrose and 10 mM HEPES, pH 7.4.

As previously described [45], isolated mitochondria (2–3 mg protein) were subjected to “aging” by incubation for 20–25 min at room temperature in 0.5 mL of a medium. This medium consisted of 65 mM KCl, 10 mM HEPES, and 1 mM KH_2_PO_4_, pH 7.4.

The fatty acid (FA) composition of liver mitochondrial membranes was analyzed using gas chromatography and mass spectrometry. Acidic methanolysis of mitochondrial membrane lipids produced fatty acid methyl esters (FAMEs) [25,26]. After extraction with hexane, the FAMEs solutions were analyzed. For FAME quantification, a Kristall 2000 M chromatograph was used, featuring a flame-ionization detector and a DB-1 quartz capillary column (60 m × 0.32 mm, 0.25 µm phase film) sourced from J&W Scientific (Agilent Technologies, Folsom, CA, USA). The FAME analysis employed a temperature program, increasing from 120 °C to 270 °C at a rate of 4 °C/min. Quantification of FAME in samples involved calculating the proportion of each acid’s peak area relative to the aggregate peak area of all identified FAMEs [46]. The variability of average peak areas, derived from triplicate measurements, remained within a 5% relative standard deviation. FAME identification in the samples was achieved through mass spectrometry, following separation under conditions mirroring the gas chromatographic analysis, utilizing a Hewlett Packard 6890 instrument (Hewlett-Packard, Palo Alto, CA, USA). Mass spectra were generated via electron impact ionization at 70 eV, with a scan rate of 1 cycle per mass decade across the 40–400 Dalton range.

The protective activity of AMB was studied in Balb/c mice using acute hypobaric hypoxia models and forced swimming tests.

Acute hypobaric hypoxic conditions were simulated in the mice in a hypobaric chamber at a pressure of 230.40 mmHg, simulating an altitude of 9000 m above sea level. A vacuum equivalent to 5000 m altitude (atmospheric pressure 405 mm Hg) was created for the first minute. Then, the “ascent” continued at a rate of 1000 m/min. The mice stayed at the simulated 9000 m altitude for 5 min.

In the forced swim test, mice were placed in room-temperature water with each mouse carrying a weight representing 2% of its body mass [47].

The Student’s *t*-test was used for statistical analysis of the results. The experimental results are presented as mean values with standard deviations. Differences between variants were considered statistically significant at a *p* value of ≤0.05. Results were processed using Microsoft Excel and SigmaPlot 10 software.

## 5. Conclusions

Ambiol’s ability to maintain initial (control) levels of C_18_ and C_20_ unsaturated fatty acids appears to protect against stress-induced mitochondrial dysfunction, thereby supporting cellular energy metabolism, which likely increases the body’s resistance to stress.

## Figures and Tables

**Table 1:** The influence of Ambiol and AHH on the mean liver mitochondrial volume in mice, as determined by atomic force microscopy.

Group	Average Volume, μm^3^	−0.95	+0.95
Control	0.243	0.202	0.284
AHH	0.534	0.497	0.575
Control + AMB	0.256	0.226	0.285
AHH + AMB	0.274	0.247	0.283

**Table 2:** The rate of NAD-dependent substrate oxidation by liver mitochondria (ng_·_ mol. O_2_/mg protein × min). Data from ten biological experiments are presented.

Group	V_2_	V_3_	V_4_	V_3_/V_4_	V_FCCP_
Control	7.8 ± 1.2	36.2 ± 1.3	9.28 ± 0.50	3.90 ± 0.03	37.1 ± 1.1
AHH	8.9 ± 1.4	25.3 ± 1.4	10.50 ± 0.30	2.40 ± 0.02	26.4 ± 1.4
AHH + AMB	7.5 ± 1.0	37.23 ± 1.5	9.33 ± 0.50	4.00 ± 0.05	39.5 ± 1.2

**Table 3:** Oxidation rates (ng·mol. O_2_/mg protein × min) of NAD-dependent substrates as influenced by different AMB concentrations, were derived from 10 biological experiments.

Ambiol, M	Vo	V_3_	V_4_	V_3_/V_4_	FCCP
-	7.1 ± 0.9	28.9 ± 2.0	9.6 ± 1.0	3.00 ± 0.03	32.9 ± 1.5
10^−5^	8.1 ± 1.2	45.0 ± 2.3	11.7 ± 1.0	3.85 ± 0.04	50.5 ± 1.5
10^−6^	7.5 ± 1.4	43.7 ± 1.5	12.1 ± 0.9	3.61 ± 0.05	43.8 ± 1.3
10^−7^	6.1 ± 1.2	30.5 ± 3.3	10.5 ±1.3	2.90 ± 0.02	33.2 ± 1.7
10^−9^	7.3 ± 1.0	37.6 ± 1.1	11.8 ± 1.4	3.20 ± 0.04	39.2 ± 1.9
10^−12^	6.5 ± 1.3	22.7 ± 0.9	9.5 ± 1.0	2.38 ± 0.03	26.0 ± 1.7

Incubation medium and Legend are the same as in Table 2.

## Data Availability

The original contributions presented in this study are included in the article/Supplementary Materials. Further inquiries can be directed to the corresponding author.

## Supplementary Materials

The following supporting information can be downloaded at: https://www.mdpi.com/article/10.3390/ijms27083589/s1.

## Abbreviations

The following abbreviations are used in this manuscript:

ROS	reactive oxygen species
LPO	lipid peroxidation
Fas	fatty acids
FAMEs	fatty acid methyl esters
AMB	Ambiol
