# Supplementary material for: Ambiol Prevents Changes in the Functional Characteristics of Mitochondria Under Hypoxia
Source: Int J Mol Sci. 2026 Apr 17;27(8):3589. doi: 10.3390/ijms27083589 (PMC13116641; doi:10.3390/ijms27083589)
Supplement: Supplementary file 1 [file ijms-27-03589-s001.zip › ijms-4230979-supplementary.pdf]

Supplementary Materials:

Study of Mitochondrial Volume Using Atomic Force Microscopy

Mitochondrial volume was assessed using atomic force microscopy. Mitochondrial samples were prepared by fixing them with a 2% glutaraldehyde solution for one hour, then washed with water and centrifuged to remove excess liquid. The next step was the deposition of mitochondria on a silicon substrate and then air-dry them. The SOLVER P47 SMENA device operating at a frequency of 150 kHz in semi-contact mode was used for the study. The analysis was performed using an NSG11 cantilever with a radius of curvature of 10 nm. Some geometric parameters of the mitochondrial image were determined using the Image Analysis software. The cross-section of the images was taken at a height of 30 nm. The volume of the mitochondrial image corresponded to the product of the cross-sectional area of the mitochondrial image and the average height of this image in the cross-sectional area**.**

Acute Cytotoxic Hypoxia Model

Modeling of cytotoxic hypoxia was carried out by intraperitoneal injection of 20 milligrams of sodium azide per kilogram of body weight

Model of acute hypobaric hypoxia

Modeling of acute hypobaric hypoxia in mice was carried out in a hyperbaric chamber in a low-pressure atmosphere (230.40 mm Hg. tbsp.), which corresponds to the height of 9000 m above sea level. During the first minutes the rarefaction corresponding to 5 thousand meters (corresponding to the atmospheric pressure of 405 mm Hg. tbsp.) above sea level was created in the chamber. In each subsequent minute "ascent" was carried out on the further one thousand meters. The residence time of mice "at a height" of 9.0 thousand meters above sea level - was 5.0 minutes

Table S1. Protective activity of Ambiol (Results of 10 biological experiments are presented).

Exposure	Measured parameter	Control	AMB
		0	10^{-6}mol/kg
"Ascent" to a height of 11.5 thousand meters (hypobaric hypoxia)	Lifetime in minutes<br>% of survivors	4,0±1,2<br>20%	14,5± 2,0<br>40%
Sodium azide injection 20 mg/kg (cytotoxic hypoxia	Lifetime in minutes<br>% of survivors	5,1±0,9<br>20%	20,3±1,4<br>50%
Swimming with a load	Lifetime in minutes<br>% of survivors	10,6±1,3<br>20%	21,4±1,5<br>38%

Research Involving Animals

The research was performed in accordance with the approved protocol and standard operating procedures of the researcher (SOPR), as well as with the Guidelines for laboratory

animals and alternative models in biomedical research on laboratory animals [46]. Our study was conducted on the topic approved by the Ministry of Science and Higher Education of the Russian Federation (minobrnauki.gov.ru): 44.4. A comprehensive study of the mechanisms and effects of the action of natural and synthetic antioxidants, antitumor drugs, chemical and physical factors. The study of biological aging mechanisms. Development of new methods of therapy and diagnosis of socially significant diseases, number 122041300205-8. On this topic, work is carried out with permitted laboratory animals (mice) on an ongoing basis under the supervision of the Ethics Committee of the Emanuel Institute of Biochemical Physics, Russian Academy of Sciences. It is carried out in accordance with the requirements for work using laboratory animals and international recommendations for biomedical research (CIOMS) and other regulatory documents on this problem.
